# A Demonstration of the Nerves of the Human Body. Part III. Cerebral Nerves

**Published:** 1833-04-01

**Authors:** 


### A Demonstration of the Nerves of the Human Body. Consisting of Four Parts. Part III. The Cerebral Nerves. By *Joseph Swan*. Price three guineas and a half.

We beg to solicit public attention to this splendid work. There are occasions on which the language of consistent approbation would seem to be mere encomium, and this is one. The work of Mr. Swan is creditable not only to himself, but to his country; a memorable evidence of spirit and industry, and a splendid specimen of art. There is no other country in which such anatomical engravings have been published, or perhaps in which they could be published, and it is melancholy to reflect, that even here they must be, comparatively, published in vain. It is impossible for professional men to purchase out of their scanty earnings such magnificent and necessarily costly books. Mr. Swan knows this, and knowing it, perseveres: a rare instance of noble public spirit, and an evidence of a generous and manly mind. Mr. Swan is content to labour for reputation, and to incur a sacrifice both certain and severe without even a faint hope of pecuniary reimbursement.

Were our medical corporations such as they should be, were they organized for professional advantage and general good, they might patronize with effect undertakings of this description. The College of Surgeons should superintend the production of a system of anatomical engravings which should be of real service to anatomy, and raise the scientific character of the nation. The greater part of the profession have to struggle for the means of existence, and they cannot embark in speculations which entail pecuniary loss, nor have they the power of encouraging those who do embark in them. Even they who have better prospects and look to fairer days are compelled, by the state of society, to direct their attention to that which is calculated to increase their chances of obtaining wealth. We need scarcely say, that anatomical researches are not so calculated.

However the facilities for the prosecution of human anatomy may be augmented, there is still a strong necessity for anatomical plates. If this be granted, and the demand for such plates does, we think, render argument useless, it necessarily follows, that the better they are, the more satisfactorily will they fulfil their objects. But individuals must labour for reward; there are few so public-spirited as to supply a public want at a personal risk or a private loss. The consequence is, that those who attempt to meet the demand are induced to offer cheap and inferior articles. It is only a public body which could command the means or would incur the risk of supplying what is wanted. We do not however hesitate to say, that our College of Surgeons should superintend the publication of a series of anatomical plates, a series which should compete with these Demonstrations of the Nerves; nay, more, we do not hesitate to express our belief, that if such a plan were adopted by the College, with the utmost possible economy, consistent with the end in view, with liberality in the design, and cheapness and convenience in the execution, no loss would accrue to itself and an infinite gain and much satisfaction to the profession.

It is too generally believed, and there is no utility in blinking the matter, that the College has existed and does exist too much for itself and too little for the body politic. It is easy to foresee that changes in its constitution are impending, and it is equally easy to conceive that such changes must lead to measures of more general benefit than have hitherto emanated from the Corporation. Among such measures we would certainly place, the execution of such designs as those we have alluded to, designs too extensive to be carried into effect by private individuals, yet not too vast for corporate associations.

We say it is easy to foresee that a change is impending over our Colleges. They are not what they have been, they are not what they will be. Those changes we cannot pretend to specify, but of this we may be sure, that their aim *must* be to render the establishments more suited to the spirit of the day, and the exigencies of the profession. Colleges in fact we have none. Three Corporations there are, but they are no more the Colleges of the profession than of the order of the Jesuits. The officers and the members, the notables and the tiers état, are like Nebuchadnezzar's image, iron and clay, things which may by force be soldered, but can never be amalgamated. Nay, even the very soldering is gone, and, as in some old building, time and the weather have acted on the *clamps*, and the necessary chemical action of the discordant materials on each other has loosened the hold and destroyed the connexion. We trust that the Colleges will reform from within before they are struck from without. We trust and indeed we believe that there are those amongst the Councils and Committees who can see clearly and resolve wisely, and whose discernment and decision will be productive of benefit to all.

It is not our present purpose to write an article on medical reform, but we were led not unnaturally to indulge in these reflections, on contemplating the splendid work before us, a memorial at once of private enterprise and private loss. We will desist.

The Part before us contains seven Plates of the Cerebral Nerves. It consists of a description of the nerves and a copper-plate engraving or engravings, accompanied with a correspondent outline and explanatory letter-press, representing each nerve in as many points of view as its character requires. As a work of art it possesses merit of the highest order, but its merit is equally great, if we may be pardoned the antithesis, as a work of nature. We hardly know which we should the most admire, the skill of the dissector who prepared, or of the draughtsman who delineated. If true anatomy can be learnt from plates it would be learnt from these, and he must be a narrow-minded thinker who could dream, after looking at these, that anatomical plates are injurious. When the memory shall convey images as definitely and as permanently as the graver, then, and not till then, will good anatomical plates be unnecessary. Are maps of no service to those who have traversed or who are to traverse an intricate country?

Were we to select engravings of particular beauty we should pitch upon those of the fifth nerve, and of the portio dura of the seventh. But the delineations of the nerves of the tongue and of the pharynx are almost equally beautiful and must have been much more difficult. We must speak as favourably of the descriptive portion; in short, it is a work which transforms criticism into eulogy, and a critic into an advertiser. We suffer the metamorphosis most willingly, and happy should we be could we induce our readers to encourage the work by subscribing to it, or rather, could we give them the means of following what must be their inclinations. Book societies and the schools of medicine should expend their patronage on what is truly a national publication.

---

